# Analog spatiotemporal feature extraction for cognitive radio-frequency sensing with integrated photonics

**DOI:** 10.1038/s41377-024-01390-9

**Published:** 2024-02-14

**Authors:** Shaofu Xu, Binshuo Liu, Sicheng Yi, Jing Wang, Weiwen Zou

**Affiliations:** State Key Laboratory of Advanced Optical Communication Systems and Networks, Intelligent Microwave Lightwave Integration Innovation Center (imLic), Department of Electronic Engineering, Shanghai Jiao Tong University, Shanghai, China

**Keywords:** Optoelectronic devices and components, Microwave photonics

## Abstract

Analog feature extraction (AFE) is an appealing strategy for low-latency and efficient cognitive sensing systems since key features are much sparser than the Nyquist-sampled data. However, applying AFE to broadband radio-frequency (RF) scenarios is challenging due to the bandwidth and programmability bottlenecks of analog electronic circuitry. Here, we introduce a photonics-based scheme that extracts spatiotemporal features from broadband RF signals in the analog domain. The feature extractor structure inspired by convolutional neural networks is implemented on integrated photonic circuits to process RF signals from multiple antennas, extracting valid features from both temporal and spatial dimensions. Because of the tunability of the photonic devices, the photonic spatiotemporal feature extractor is trainable, which enhances the validity of the extracted features. Moreover, a digital-analog-hybrid transfer learning method is proposed for the effective and low-cost training of the photonic feature extractor. To validate our scheme, we demonstrate a radar target recognition task with a 4-GHz instantaneous bandwidth. Experimental results indicate that the photonic analog feature extractor tackles broadband RF signals and reduces the sampling rate of analog-to-digital converters to 1/4 of the Nyquist sampling while maintaining a high target recognition accuracy of 97.5%. Our scheme offers a promising path for exploiting the AFE strategy in the realm of cognitive RF sensing, with the potential to contribute to the efficient signal processing involved in applications such as autonomous driving, robotics, and smart factories.

## Introduction

Fast, accurate, and low-cost interpretation of valid information from raw signals is a fundamental capability for cognitive sensing systems such as unmanned vehicles, robots, and smart factories^1–3^. Fully digital systems are designed to achieve this goal by digitally sampling all received signals at high fidelity and extracting information with digital signal processors (DSPs). While this strategy is flexible and accurate, it overlooks the intrinsic sparsity of critical information^4,5^. In situations with broadband signals and multi-channel transceivers, the fully digital strategy can produce a great deal of redundant data, occupying a significant amount of memory and processing resources, due to the slow “information rate” compared to the signal sampling rate. This puts undue stress on high-speed analog-to-digital converters (ADCs) and high-performance digital signal processors (DSPs), leading to costs that exceed the affordable limit of mobile or distributed sensing platforms. Instead, the analog feature extraction (AFE) strategy transforms raw signals into sparse features in the analog domain, significantly reducing the data rate of backend ADCs and digital processing while maintaining accuracy^6,7^. AFE is typically performed in real-time, and the digital backend processes only a small amount of data, making it more efficient and lower latency than a fully digital strategy. Recently, the AFE strategy has successfully tackled narrowband tasks including voice, electrocardiogram, and electroencephalogram^8–11^, showing latency and power consumption advantages. However, in the realm of RF sensing and detection, a large instantaneous bandwidth over several GHz is typically required to achieve a good resolution of target details^12,13^. The electronic RF circuit’s bandwidth and reconfigurability bottlenecks have hindered the ability to extend the advantages of AFE to broadband cognitive RF sensing applications^14,15^.

Photonics is considered a competitive candidate for RF signal processing due to its broadband capability and reconfigurability. The development of microwave photonics technologies has facilitated the generation, filtering, receiving, and processing of signals with instantaneous bandwidth over several GHz across the frequency range of tens of GHz^16–20^. Furthermore, photonic integration has enabled the creation of complicated programmable processors^21–23^. Hence, emerging photonic neural network (PNN) accelerators harness photonic devices to construct high-performance artificial intelligence processors that excel in terms of clock rate, throughput, and power efficiency^24–28^. The PNN accelerators process signals in the analog domain, and a variety of neural network models, including fully connected^29–33^, convolutional^34–37^, and recurrent^38^, have been successfully demonstrated. For machine vision tasks, PNN accelerators are used to process optical images directly in the analog domain^39–41^. The latency of image classification can be reduced to 570 ps, indicating the latency advantage of photonic analog processing. Recently, a PNN is adopted as the frontend of an RF blind source separation system^42^. It supports an operation bandwidth over 19.2 GHz, showing a great potential of using photonics to process broadband RF signals. However, this work processes the RF signals in the spatial dimension. It cannot conduct the cognitive RF sensing tasks which require both spatial and temporal information. Although a photonic tensor convolutional processor capable of processing signals from both spatial and temporal dimensions was recently proposed^43^, to the best of our knowledge, the photonic AFE for broadband RF applications has rarely been investigated.

Here, we introduce a photonic analog feature extraction (PAFE) concept for cognitive RF sensing systems, which uses an integrated photonic circuit as the feature extractor. The photonic circuit converts the broadband RF signals into sparse features. Therefore, low-speed ADCs are capable of recording the features and the computing resource occupied by backend processing is significantly reduced. The photonic feature extractor with tunable components can be trained to match the application-specific feature requirements. Furthermore, the photonic circuit manipulates the received waveforms from multiple spatially distributed antennas, which enables extracting features from the spatial-temporal-joint dimension. The validity of features is thus enhanced compared with single-dimension feature extraction. We further propose an analog-digital-hybrid transfer learning (ADT-learning) scheme for low-cost and effective training of the photonic circuit. The PAFE concept is validated with a high-resolution radar target recognition task. Experimental and analytical results verify that critical spatiotemporal features are effectively extracted with a 4-time reduced sampling rate and the targets are successfully recognized with an accuracy of 97.5%.

## Results

### Photonic analog spatiotemporal feature extraction

**Fig. 1:**
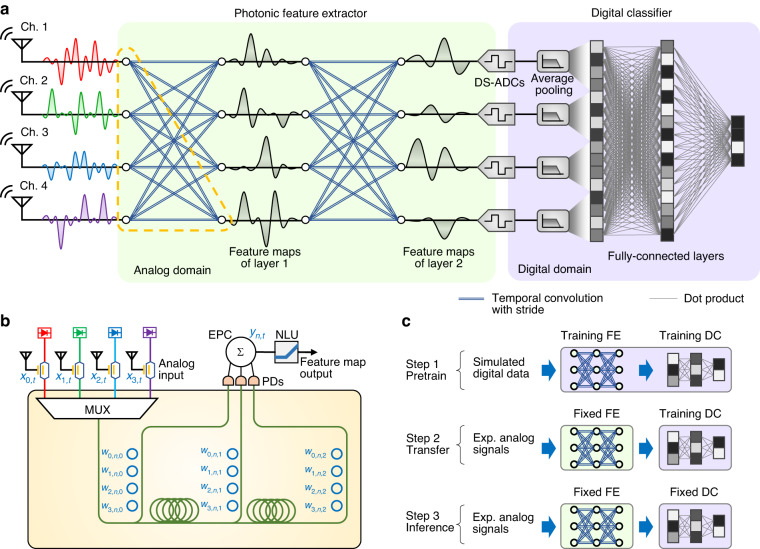
Conceptual schematics of the PAFE. **a** Architecture of analog spatiotemporal feature extraction and digital classification. The raw waveforms from the multichannel RF front end are processed by the photonic feature extractor in the analog domain. A blue double line represents a temporal convolution with strides. After several convolutional layers, the feature maps are digitally recorded by DS-ADCs. Fully connected layers are built in the digital domain as the classifier. **b** An example of the hardware implementation of the PAFE concept. **c** The schematic of ADT-learning. The first step pretraining is fully digital for training the feature extractor (FE). The second step uses the trained feature extractor in the analog domain to acquire a dataset composed of feature maps. Then retrain the digital classifier (DC). The third step is inference with the trained analog feature extractor and the retrained digital classifier

The PAFE is based on the concept of a convolutional neural network (CNN), as shown in Fig. 1a. Typically, a CNN adopts multiple convolutional layers as the feature extractor and several fully connected layers to perform classification. In each convolutional layer, multiple input channels are convolved by different sets of convolutional kernels, and feature maps are obtained at the output. Nonlinear activation and pooling layers are then used to increase the network’s nonlinear fitting capability. Layer by layer, the original input data is transformed into high-level sparse features that can be classified by the fully connected layers. In the PAFE scheme, the analog photonic circuit performs the convolutional feature extraction, while classification is accomplished using digital electronics. The original signals received from the RF frontend are modulated onto optical carriers and directly fed into the photonic feature extractor. They are first convolved in the temporal dimension with different kernels to yield multiple outputs, which are then accumulated to obtain the feature maps. Therefore, the feature maps include information from both the temporal and spatial dimensions. Multiple spatiotemporal feature maps are obtained simultaneously using the same steps. For each feature map, an electro-optic nonlinear activation unit (NLU) is adopted to carry out the nonlinear activation function such as the rectified linear unit (ReLU) in the analog domain^44–46^. The striding of convolution is set larger than 1, resulting in feature maps that are smaller than the input signals. In the digital domain, the striding operation can be achieved by discarding output data from a non-striding convolution. In the analog domain, striding operation is similarly realized by not recording the unwanted waveform samples at the analog-to-digital conversion. Supplementary note S1 provides details on the striding operation in the analog domain. With analog striding, the required sampling rate for recording the feature maps is lower than that for recording the original signals. After several convolutional layers, the required sampling rate for recording the feature maps is significantly reduced, so the feature maps are digitally recorded using down-sampling ADCs (DS-ADCs). In the digital backend, an average pooling layer further reduces the data rate of digital processing, and a backend algorithm is deployed in the digital processor. For classification tasks, several fully connected layers can be deployed to transform the feature maps into a one-hot vector as the classification result.

The hardware implementation of the spatiotemporal feature extraction is exemplified in Fig. 1b. It follows the principle of an integrated photonic tensor convolution processor^43^ and corresponds to one output channel, represented by the yellow-dashed-circle-marked box in Fig. 1a. Replicating this structure multiple times can output multi-channel feature maps. Firstly, the analog signals are modulated onto optical carriers of different wavelengths and multiplexed into a single optical waveguide. Following this, a group of delay lines applies uniform time delays to the input signals. For each delay step, a micro-ring weighting bank is deployed to multiply convolutional weights to the input signals. The number of microrings in a weighting bank equals the number of wavelengths, with each microring tuned to modulate the transmission rate of a specific wavelength. As a result, the signals of different wavelengths and delay steps are specifically modulated. Finally, photodetectors (PDs) are employed to convert the optical signals to electrical signals and sum the power of all wavelengths. An electronic power combiner (EPC) then accumulates the electrical signals of all delay steps. The multiply and accumulation process of the photonic tensor convolutional processor is formulated as follows.

1 $${y}_{n,t}=\mathop{\sum }\limits_{m=0}^{M-1}\mathop{\sum }\limits_{i=0}^{\sigma -1}{w}_{m,n,i}\cdot {x}_{m,t+i\cdot \tau }$$

where *x*_*m*,*t*_ is the input signal of the *m*-th channel, *w*_*m*,*n*,*i*_ is the convolutional kernel, and *y*_*n*,*t*_ is the output signal of the *n*-th channel. As the first accumulation represents the temporal convolution with the kernel width of *σ* and the second accumulation suggests spatial information synthesis across the *M* input channels, the output signal yields a spatiotemporal feature map. The feature map is activated by a subsequent nonlinear unit (NLU) and enters the next convolutional layer for deeper feature extraction. As long as one output channel is implemented, integrating more weighting banks on the chip can calculate the complete convolutional layer, which is discussed in refs. ^43,47^.

A valid feature extractor must be obtained through proper training of the convolutional kernels. However, it can be challenging and costly to train the photonic feature extractor conventionally with digital computers and digital data in the context of the PAFE scheme. Training the PAFE requires raw signals from the RF frontend captured in digital format, which necessitates the deployment of an expensive high-speed sampling system solely for PAFE parameter training. Moreover, constructing a sufficient training dataset demands a large number of examples acquired in numerous scenarios, which is difficult to achieve in the real world due to the expense of a high-speed multichannel RF sampling system and the unacceptable effort of building such plentiful sensing scenarios. To address this problem efficiently, we propose an analog-digital-hybrid transfer learning (ADT-learning) scheme. This scheme relies on a three-step process illustrated in Fig. 1c. The first step is pretraining with fully digital data generated by simulation. A CNN model that is identical to the PAFE model is trained with the simulated dataset. As long as the simulation is similar to the practical scenarios, the pretraining will obtain a valid feature extractor. Then, the parameters of the feature extractor are fixed. The second step involves transfer learning, where the trained feature extractor’s parameters are loaded onto the photonic feature extractor that extracts analog feature maps. Practical scenarios are built for capturing these feature maps. Only the final feature maps are digitally recorded with the DS-ADCs. During transfer learning, the classifier is retrained with the experimentally recorded analog feature maps, which reasonably compensate for the gaps between the simulated and practical scenarios and between the digital CNN and photonic circuit models. The third step is using the trained analog feature extractor and the retrained digital classifier for inference. Typically, a dataset for transfer learning can be much smaller than that of conventional training. The effort and cost to build scenarios are significantly reduced. It makes the ADT-learning scheme a feasible approach to well-train analog feature extractors.

### High-resolution radar target recognition

**Fig. 2:**
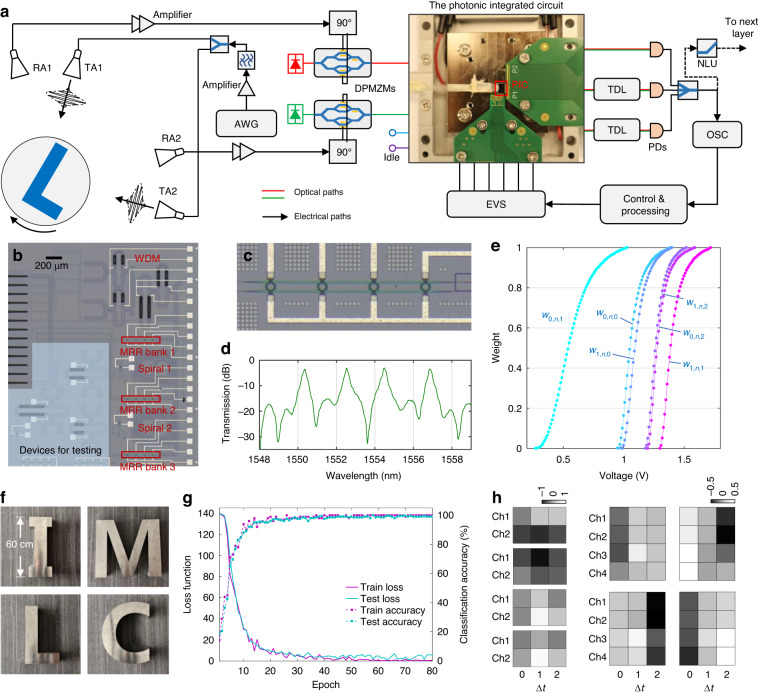
Experimental setup of high-resolution radar target recognition. **a** Experimental setup of the first convolutional layer based on the integrated photonic tensor convolutional processor. The RF pulse signal is generated by an arbitrary waveform generator (AWG) and is split into two transmitting antennas (TAs). The echoes are received by the receiving antennas (RAs) and amplified. Ninety-degree hybrid (labeled with ‘90°’) is used to drive the DPMZM working at carrier-suppressed single band mode. The tunable fiber delay lines (TDLs) are used to realize analog convolutions with strides. Oscilloscope (OSC) records the intermediate feature maps. The electrical voltage source (EVS) array sets voltages to the microrings to set weighting parameters. **b** The photograph of the fabricated photonic integrated circuit. Its layout is the same as the schematic of Fig. 1b. **c** The zoom-in photograph of the MRR weighting bank. **d** The original resonance wavelengths of an MRR weighting bank. **e** The transmission-voltage curves of the adopted MRRs. Their corresponding weights are labeled. **f** Photographs of the reflective targets adopted for recognition. “I”, “M”, “L”, and “C” with a height of 60 cm. **g** The results of pretraining. **h** The trained convolutional kernels. The first column shows four 2 × 3 kernels of the first layer. The second and third columns show four 4 × 3 kernels of the second layer

The photonic analog feature extractor (PAFE) is capable of tackling broadband RF signals, and thus, we demonstrate its effectiveness through a high-resolution radar target recognition task. The photonic analog feature extractor is configured as two convolutional layers, while the digital classifier consists of two fully connected layers. The first convolutional layer is composed of two input channels and four output channels with a kernel width of 3 and a stride of 2. Signals are acquired directly from the RF frontend shown in Fig. 2a, with two transceivers transmitting the same RF pulse signal with a 3-dB bandwidth of 8–12 GHz to detect the range profile of the target at different angles. The signals from both angles are modulated onto the optical carrier with dual parallel Mach-Zehnder modulators (DPMZMs) working in the carrier-suppressed single-band mode. The optical signals are then processed by the photonic integrated circuit. The adopted photonic integrated circuit is fabricated with a standard silicon-on-insulator process, shown in Fig. 2b. It has the same structure as the schematic in Fig. 1b, containing a 4-wavelength WDM, three microring resonator (MRR) weighting banks, and two spiral delay line between each weighting bank. The WDM is designed by the asymmetric Mach-Zehnder interferometer tree architecture^48^. Its channel spacing is 2 nm. An MRR weighting bank is shown in Fig. 2c, containing four thermo-optic tunable MRRs with a 10-nm free spectrum range. Their original resonance points are depicted in Fig. 2d, close to the designed 2-nm spacing. In order to apply weights to the MRRs, we measured their transmission-voltage curves as shown in Fig. 2e. Because of the fabrication error, we applied different offset voltages to these MRRs to shift their resonance points to the adopted wavelengths (1550.8 nm and 1552.8 nm). With these curves, the EVS generates corresponding voltages to apply weights.

As a proof-of-concept, we employ the photonic integrated circuit repeatedly to complete the feature extractor. Details of the photonic circuit reusing are described in the Methods. The output of the photonic integrated circuit corresponds to the spatiotemporal feature map of one output channel. Tuning the parameters on the photonic circuit and repeating the experiment allows for the acquisition of other feature maps of the first convolutional layer. The analog feature maps of the first layer should undergo nonlinear activation and be sent to the next layer for further feature extraction. In the experiment, an oscilloscope is used to observe and temporarily record the intermediate feature maps. Four intermediate feature maps of the first layer are recorded digitally so that we can reuse the same photonic circuit to handle the second convolutional layer. A digital nonlinear activation is conducted on the recorded intermediate feature maps to imitate an electro-optic nonlinear unit. We should note that a complete PAFE system (shown as Fig. 1a) does not require digital components to record the intermediate feature maps. Supplementary note S2 discusses the feasibility of implementing a complete PAFE system. The delay unit of the first layer is 200 ps, which represents a sampling rate of 5 GSa/s and fulfills the Nyquist bandpass sampling of the 8-12 GHz pulse echoes. Here, tunable delay lines (TDLs) are used after the photonic circuit to precisely adjust the relative delay amount of the output ports. For the second convolutional layer, the number of input channels and output channels is set to 4. The kernel width is 3, and the stride is 2. The activated feature maps from the first layer are generated by the arbitrary waveform generator (AWG), modulated on the optical carriers with DPMZMs operating in intensity-modulation mode and convolved by the photonic circuit. Considering that the stride of the first layer is 2, the delay unit for the second layer is 400 ps to avoid processing unwanted waveform points. TDLs are again used to setup the 400-ps relative delay amount. With the experimental setup described above, the analog feature extractor is conducted. The final feature maps are digitally recorded via DS-ADCs at the sampling rate of 1.25 GSa/s (800-ps time interval) to discard the unwanted waveform points of the second convolutional layer. Hence, the sampling rate of the DS-ADC is one-fourth of that required by Nyquist sampling the original RF signals.

Figure 2f displays four aluminum reflective targets with distinct shapes (“I”, “M”, “L”, and “C”), utilized for the target recognition task, with the objective of correctly classifying the targets at various observing angles. To achieve this, the analog feature extractor parameters were trained based on ADT learning. The pretraining process is explained in the Methods section, and the results of pretraining are depicted in Fig. 2g. The loss functions of training and testing drop consistently and the recognition accuracies increase consistently, demonstrating successful training. The average testing accuracy reaches 99.5% in the pretraining. Figure 2h presents the trained parameters of convolutional kernels that show structures capable of edge detection, smoothing, and other patterns across both temporal and spatial dimensions when working on the inputs, implicitly suggesting the efficient extraction of spatiotemporal features.

**Fig. 3:**
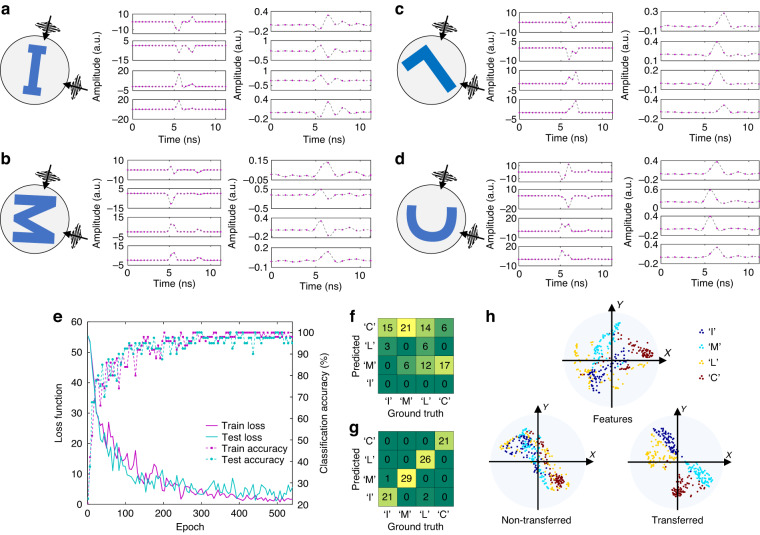
Experimental results of PAFE and radar target recognition. **a**–**d** Examples of the extracted feature maps of the target of “I”, “M”, “L”, and “C”. For each target, the first column shows four intermediate feature maps of the first convolutional layer and the second column shows four final feature maps of the second convolutional layer. Markers on the plot are the samples at 2.5 GSa/s and 1.25 GSa/s, respectively. **e** Loss functions and classification accuracy of transfer learning. **f**, **g** The confusion matrices of classification before and after transfer learning. **h** Result of t-SNE for the final feature maps, non-transferred classifier, and the transferred classifier, respectively

With the pre-trained convolutional kernels, we acquire the feature maps of the target using the photonic circuit. Several examples are shown in Fig. 3a–d. The feature maps of convolutional layer 1 help us analyze how the CNN recognizes the targets. Different target shapes result in differing numbers of scatters, with “M” and “C” producing more sub-echoes with more peaks on the feature maps than other shapes. Additionally, peak locations vary by shape, which may be a crucial element in differentiating between “I” and “L”. However, the feature maps of the second layer are more abstract and incomprehensible. The final feature maps are then used for transfer learning to fine-tune the digital classifier, which significantly increases classification accuracy to 97.5% on average, from the result depicted in Fig. 3e. Figure 3f, g show the classification results before and after transfer learning, respectively. Without transfer learning, the classification accuracy is even lower than the accuracy of random guesses. It indicates that the inconsistency between the digital CNN model and the analog feature extractor and the inconsistency between the simulated echoes and the experimental echoes have a substantial impact on the pre-trained classifier. Hence, the transfer-learning is critical for the digital classifier to achieve high-accuracy recognition.

To ascertain the validity of the analog feature extractor, we apply the t-stochastic neighbor embedding (t-SNE)^49^ as the dimensionality-decreasing tool to inspect the features extracted by the photonic circuit. From Fig. 3h we observe that the extracted features of every specific target tend to gather. Although the features cannot be directly separated, they can become linearly separatable with the nonlinear transformation of the fully connected layers. However, the non-transferred classifier fails to transform features in the correct way such that they remain non-distinguishable. The transferred classifier successfully transforms them into linearly separable features, leading to high classification accuracy. Consequently, the results demonstrate that the analog feature extractor can obtain valid features for high-accuracy classification, and correctly transferred digital classifiers yield accurate classification results.

**Fig. 4:**
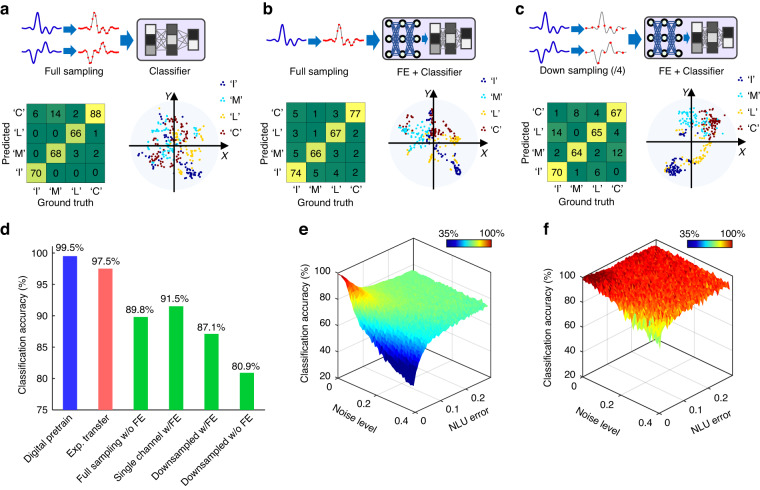
Results of the comparison study for evaluating the effectiveness and functionality of the PAFE. **a**–**c** The confusion matrix and the t-SNE map of the neural networks with different configurations. **a** The input is two-channel Nyquist-sampled data but the neural network does not have a convolutional feature extractor. The classifier is the same as the one adopted in the PAFE experiment. **b** The input is single-channel Nyquist-sampled data and the neural network has the same structure as the PAFE experiment. Since the lack of spatial information, the neural network classifies the targets based on only temporal information. **c** The input is directly 4-time down-sampled data and the neural network has the same structure as the PAFE experiment. **d** The comparison of classification accuracies of different configurations. The “digital pretrain” and the “exp. transfer” are the results of Figs. 2g and 3e. “Full sampling w/o FE”, “single channel w/ FE”, and “downsampled w/FE” list the accuracies of subfigures **a**–**c**, respectively. “Downsampled w/o FE” is the accuracy based on down-sampled data and a neural network without convolutional layers. **e**, **f** The PAFE performance with respect to the noise and the NLU error before and after transfer learning, respectively

We conducted a comparison study with various neural network configurations to evaluate the effectiveness and functionality of the PAFE. Figure 4a–c depict the confusion matrices and t-SNE results of three configurations with different input data down-sampling techniques and neural network structures: (1) Two-channel Nyquist-sampled input data and only a classifier, (2) Single-channel Nyquist-sampled input data and a full CNN, and (3) Two-channel down-sampled input data and a full CNN. Figure 4d shows their classification accuracies, which are notably lower than those achieved by the PAFE. The t-SNE results show that neural networks failed to distinguish the targets under these three configurations. Comparing Configuration (1) to the PAFE reveals that the convolutional feature extractor is indispensable to extract critical information of the target echoes. The single-channel data of Configuration (2) does not carry sufficient information to distinguish targets, despite its complete neural network. PAFE’s capability of extracting spatial-temporal-joint features is a vital factor in obtaining good recognition performance. Finally, the result of configuration (3) indicates that critical information loss may occur if the input echoes are directly down-sampled instead of down-sampled by a feature extractor. Therefore, the numerical study confirms that the PAFE extracts spatiotemporal features through convolving multi-channel input signals, resulting in accurate recognition with down-sampled digital data.

## Discussion

As an analog computing strategy, the performance of PAFE is subject to analog errors, primarily from noise, weight deviation, and nonideal electro-optic NLUs. Feedback-controlling methods offer a way to achieve high-precision weights over 9 bits^50^. Therefore, we conducted an analysis of the impact of random noise and nonideal electro-optic NLUs on the performance of PAFE, as illustrated in Fig. 4e, f. Further details of the analysis can be found in Supplementary Note S3. Because the parameters loaded onto the photonic feature extractor were obtained from pretraining using simulated data and a digital CNN model, random noise and nonideal NLU of the photonic feature extractor deteriorate the quality of the extracted feature maps. Figure 4e illustrates how such deterioration significantly reduces the accuracy of digital classification without transfer learning. With minor NLU errors, the random noise consistently worsens the accuracy. As the NLU error increases, although the influence of the random noise diminishes, the accuracy decreases to roughly 70%. Figure 4f shows that after transfer learning, classification performance is enhanced to over 95% accuracy in most cases. This suggests that transfer learning can compensate for minor inconsistencies between analog NLU and digital NLU. Thus, although the NLU in our proof-of-concept experiment was implemented with a digital processor, the PAFE concept would be effective with analog electro-optic nonlinear units. Considering that a complete PAFE system should be implemented with future engineering works, Supplementary note S2 discusses the engineering feasibilities for a complete PAFE system. Also, we conducted a demonstration of a small-size two-layer PAFE system, where all linear and nonlinear operations are carried out by analog devices in real-time, as a part of our proof-of-concept experiment. Supplementary note S4 describes the details and results of this demonstration. It verifies the feasibility of a multi-layer spatiotemporal feature extractor and the reduction of ADC sampling rate.

To leverage the advantages of the AFE strategy in cognitive RF sensing systems, we propose the PAFE concept based on integrated photonics. The PAFE processes the analog signals directly from the RF frontend, with the photonic circuit playing the role of convolutional layers in a CNN to transform the raw input signals into valid spatiotemporal features. These sparse features allow the use of down-sampling ADCs to record the extracted features, instead of Nyquist sampling. Moreover, we propose the ADT-learning method for efficient training of the analog feature extractor and the digital classifier without introducing extra training effort or changing hardware configuration. In our proof-of-concept experiment, a high-resolution radar target recognition task is demonstrated to validate the PAFE concept. The sampling rate of the ADCs is reduced by 4 times compared with the Nyquist sampling while the classification accuracy reaches 97.5%. Numerical analysis verifies the effectiveness of the PAFE and the ADT-learning method for extracting valid sparse features. We believe that our proposal will catalyze the development of naturally-efficient AFE strategies for broadband RF signal processing, and provide a promising path for the next-generation cognitive RF sensing systems.

## Materials and methods

### Experimental setup

The radar-transmitted signal is Gaussian pulses so that the echoes can be directly processed with the PAFE without decoding process such as pulse compression. The signal is generated by an arbitrary waveform generator (AWG, Keysight, M8195A) and amplified by a power amplifier (Connphy, TLPA1G-18G-40-33). Two TAs transmit the same signal by adopting a microwave power splitter. For receiving, low-noise amplifiers (Connphy, TLPA1G-18G-40-33) are used to raise signal power to drive the DPMZMs (EOSPACE, IQ-0DVS-35-PFA-PFA-LB). For both transmitting and receiving, the microwave wires are strictly the same long to guarantee the same propagation length of the signals. According to the multi-input multi-output (MIMO) radar principle, a large angle among receivers usually provides a good spatial resolution. So, the first pair of TA and RA is located 90° away from the second pair. For the first layer, the DPMZMs are configured as the single-sideband carrier suppressed mode. The RF-modulated optical signal has only one sideband (shown in Fig. S7) so that it can be weighted via microrings on the photonic chip. After the photodetection, the single sideband optical signal produces a baseband electrical signal. For the second layer, the DPMZMs are configured in normal MZM mode to accept baseband electrical signals. As the signals are modulated onto the optical carrier, the integrated photonic circuit finishes spatiotemporal convolution with the assistance of TDLs, PDs, and the EPC. Because the spiral delay lines on-chip are not tunable, we adopt the TDLs (General Photonics, VDL-001-15-60-PP-FC/APC) to adjust the delay amount of each optical path so that the strided convolution of two layers is correctly realized. Strided convolution in the analog domain is further described in Suppl. Note S1. Three PDs (EOT, ET3500F) with 15-GHz bandwidth convert optical power to electric signal. The EPC is implemented with a 4-way resistive power combiner (Talent Microwave, RC4DC180SE) with bandwidth ranging from direct-current to 18 GHz. The EVS is implemented with DAC8564 and OPA172 chips mounted on a homemade printed circuit board. Its voltage resolution is better than 1 mV.

Since the integrated scale of the photonic circuit is smaller than the complete feature extractor, we reuse the photonic integrated circuit to implement the feature extractor part by part as a proof-of-concept. For the first layer with 2 input channels and 4 output channels, the photonic circuit is reused 4 times to obtain the output feature maps. The intermediate feature maps are temporarily recorded by an oscilloscope (Keysight, DSO-S) into digital data. A digital ReLU nonlinear activation function is applied to the intermediate feature maps. The second layer is designed with 4 input channels and 4 output channels. Although the fabricated chip shown in Fig. 2b supports 4 input channels at once, limited by the 2-channel AWG, the chip is operated with two input channels. The second layer is sequentially finished with 8-time reusing. Given that the adopted integrated photonic circuit can only compute positive weights, the negative weights are computed with another run. And then subtract the negative output from the positive output. Note that the proof-of-concept experiment demonstrates the proposed PAFE concept part by part, the Suppl. note S2 discusses the further engineering considerations for implementing a complete PAFE system.

### Building the pretraining dataset

The ADT-learning requires a dataset for pretraining the analog feature extractor. The stochastic characteristics of the pretraining dataset should be similar to that of the practical radar echoes so that the pre-trained feature extractor can be easily adapted to practical situations. In our experiment, the pretraining dataset is built following the sub-echoes accumulation theory of radar detection^51^. We model the shape of the targets (“I”, “M”, “L”, “C”) in a computer and then simulate the radar echoes of the targets via ray tracing, or the geometric optical (GO) method in radar books^51^, which is depicted in Fig. S8. The transmitted signal is regarded as rays and the reflected rays that enter the receiving antenna are recorded as sub-echoes. The accumulation of all sub-echoes obtains the radar echo. By rotating the targets to different angles, we collect different echoes of these targets. For each target, we collected 500 echoes from randomly selected angles, and there was a total of 2000 echoes collected for four targets. 40 randomly picked examples of the dataset are shown in Fig. S9. The dataset is randomly divided into two parts with 1600 echoes and 400 echoes, respectively. The former is the training set and the latter is the validation set. Here, we should note that although the GO method is commonly used for target modeling, it may be inaccurate when the curvature of the surface is smaller than the wavelength. In our case, the reflectivity of the target edges is much stronger than their corners, so the GO method is suitable and simplifies the modeling. If we need to achieve a much more precise pretraining or the target is rough with a feature size comparable with the RF wavelength, precise modeling methods such as FDTD should be used. In our experiment, targets with letter shapes are detected from the lateral direction. For complex targets with 3D structures, front-looking detection is feasible and the proposed spatiotemporal feature extraction concept is also applicable to these targets.

### ADT-learning

As the PAFE concept applies an analog feature extractor, the conventional training method is costly and even impractical to perform. Also, the mismatch of the digital neural network model and analog hardware model will cause severe performance degradation of conventional training. The ADT-learning method is proposed for solving the training problem of the analog feature extractor. Firstly, following the experimental setup, a CNN model is constructed on a computer. The CNN contains two convolutional layers and two fully connected layers. The convolutional kernel of the first convolutional layer has the shape of [kernel width, input channel, output channel] =[3,2,4] and the second convolutional layer has the kernel of [kernel width, input channel, output channel] =[3,4,4]. The stride for these convolutional layers is set as 2. The activation function used for convolutional layers is the ReLU function. For the fully connected layers, the number of neurons is set as 256 and 4 for the first and the second fully connected layers, respectively. The activation function is ‘tanh’ and ‘sigmoid’ for these two layers, respectively. With the above CNN model, we apply the simulated pretraining dataset to train the CNN. The trained parameters of the convolutional layers are stored as the parameters of the analog feature extractor. The second step of ADT-learning is transfer-learning. The photonic analog feature extractor collects feature maps from the experimental setup. Eighty groups of feature maps are collected for each target, and 320 groups of feature maps are available for transfer learning. In the transfer learning step, only the fully connected layers are retrained. Randomly selected 220 groups of feature maps are used for training and the remaining 100 groups are used for validation.

### Supplementary information

Supplementary Information
