# Supplementary material for: Analog spatiotemporal feature extraction for cognitive radio-frequency sensing with integrated photonics
Source: Light Sci Appl. 2024 Feb 14;13:50. doi: 10.1038/s41377-024-01390-9 (PMC10866915; doi:10.1038/s41377-024-01390-9)
Supplement: Supplementary file 1 — Supplementary Information [file 41377_2024_1390_MOESM1_ESM.docx]

**Supplementary Information for**

**Analog spatiotemporal feature extraction for cognitive radio-frequency sensing with integrated photonics**

Shaofu Xu^1^, Binshuo Liu^1^, Sicheng Yi^1^, Jing Wang^1^, and Weiwen Zou^1,*^

^1^State Key Laboratory of Advanced Optical Communication Systems and Networks, Intelligent Microwave Lightwave Integration Innovation Center (imLic), Department of Electronic Engineering, Shanghai Jiao Tong University, Shanghai 200240, China

*^*^Correspondence to:* [*wzou@sjtu.edu.cn*](mailto:wzou@sjtu.edu.cn)

Supplementary notes S1-S5;

Supplementary figures S1-S9;

Supplementary references S1-S6.

**Supplementary notes**

**S1 The realization of convolution with strides in the analog domain**

The objective of the PAFE is to extract sparse desired features while maintaining good performance in RF sensing. In convolutional neural network (CNN) models, sparse features are typically acquired through pooling operations or strided convolutions, both of which result in a reduced output size compared to the input. By employing multiple pooling layers or strided convolutional layers, the sparsity and validity of the feature maps are greatly enhanced. However, implementing nonlinear logic operations, particularly max pooling, in analog photonic circuits poses a considerable challenge. Therefore, we employ strided convolutions to increase the sparsity of the feature maps. The mathematical formulation of *s*-strided convolution is expressed as follows.

$$y_{n,l}=\sum_{m=0}^{M-1} \sum_{i=0}^{\sigma-1} w_{m,n,i}\cdot x_{m,s\cdot l+i}$$

As the index *l* of the output increases, the index *s*·*l* of the input increases *s*-time faster. Therefore, the length of the output can be *s*-time smaller than the input. Conventionally in digital computers, only the input data that matches the stride is convolved. It requires the ability of random memory access thus it is unrealizable in a photonic analog circuit.

Therefore, in this work, the strided convolution is realized by discarding (or not recording) the unwanted output data from the non-strided convolution results. Fig. S1 shows the schematic of the method. For the first convolutional layer, the delay unit is set at 200 ps to match the Nyquist sampling rate of 5 GHz. The red marks show the input data samples on an analog waveform. After the analog convolutional layer, the output waveform contains all convolution results. If the output waveform is sampled at 5 GSa s^-1^ (shown as red marks), all results are recorded so it is a non-strided convolution layer. If the output waveform is sampled at 2.5 GSa s^-1^ (marked with blue circles), only the result of 2-strided convolution is recorded. Using this method, we realize strided convolution for the first layer. For multiple layers, we change the delay unit of the following layer to match the sampling rate of the previous layer. For example, the delay unit of the second layer is configured at 400 ps to match the sampling rate of 2.5 GSa

s^-1^ of the first layer (red marks in Fig. S1b). With this configuration, the output waveform of the second layer contains all convolution results without any striding. If the waveform is sampled at 1.25 GSa s^-1^ (marked with blue circles), only the result of 2-stride convolution is recorded. If there is any subsequent layer, the delay unit should be configured larger to match the sampling rate of the previous layers, 800 ps for example.

**S2 Implementing a complete PAFE system**

The concept of PAFE consists of multiple convolutional layers implemented within an analog photonic processing system. Due to limitations imposed by experimental conditions, a proof-of-concept experiment utilizes a single photonic circuit to perform all convolutions in these convolutional layers, resulting in the demonstration of the PAFE system in a segmented manner. In order to implement a complete PAFE that can recognize complex targets in an enlarged dataset, here we deliberate on the feasibility and engineering requirements for realizing a large-scale PAFE system.

The first aspect to consider is the number of input channels. It corresponds to the number of wavelengths of a wavelength-division multiplexing (WDM) system and the number of microrings (MRRs) in each weighting bank. With a dense WDM system across the C band, 60 wavelengths are feasible [S1]. For MRR weighting banks, the number of feasible wavelengths is determined by the free spectrum range and quality factor of the MRRs. This number can theoretically exceed 100 for conventional MRRs [S2]. Therefore, the number of input channels supports large-scale integration.

Second, the number of output channels is determined solely by the number of integrated weighting banks. The input optical signals can be distributed among multiple weighting banks. Different weighting banks correspond to different output channels. Ref. [S3] discussed the feasible number of weighting banks, which can exceed 90 using commercially available photonic devices. By reducing the insertion loss of photonic circuits and utilizing sensitive avalanche photodetectors, integrating more weighting banks on-chip is possible, leading to a larger number of output channels for the PAFE system.

The third consideration is the depth of convolutional layers, because a deeper feature extractor tends to yield sparser feature maps and reduces the ADC sampling rate. However, in the case of analog convolutional layers, the depth is primarily limited by the accumulation of noise during multiple photonic-electronic inter-conversions. An in-depth analysis regarding noise accumulation and cascadability of the electro-optic nonlinear unit (NLU) link has been conducted by [S4]. Their findings indicate that the nonlinear transfer function exhibits a noise suppression effect when the nonlinear threshold is attained. By employing commercial transimpedance amplifiers (TIAs), the photocurrent from the photodetectors (PDs) can be amplified beyond the nonlinear threshold voltage within a bandwidth of 9 GHz. This observation suggests that using low-Vpi modulators, sensitive PDs, or superior TIAs makes it easier to achieve the threshold voltage, thereby increasing the depth of the photonic processor. Although stacking a deep network is theoretically feasible, it is still challenging and not necessary. Because we can utilize electronic resources to partake in some convolutional layers. The photonic feature extractor performs several convolutional layers in the front, effectively reducing the data rate. With the reduced data rate, the speed of ADCs is acceptable and the remaining part of the neural network can be quickly conducted with a digital computer. Therefore, this photonic-electronic cooperative strategy alleviates the difficulties associated with constructing deep photonic convolutional networks while simultaneously realizing AFE concept in broadband RF applications.

About the implementation of NLUs. Recent works have presented various NLU schemes exhibiting advantages such as high-speed operation, low power consumption, and profile reconfigurability. These NLUs form the foundation of a complete PAFE system. Specifically, the ReLU function adopted in the proof-of-concept experiment can be realized with lasers, MRRs, or Mach-Zehnder interferometers. Although the analog ReLU unit profile does not perfectly resemble the max{0, *x*} function, it effectively emulates the behavior of the ReLU function. The mismatch can be compensated via transfer learning, discussed in Suppl. note S3.

**S3 The mismatches of the digital network model and analog devices**

The PAFE concept builds a feature extractor in the analog domain, whose parameters should be trained properly for extracting valid features. During pretraining, a network model built in the digital computer is trained and the parameters obtained is regarded as the parameters to be loaded onto the photonic feature extractor. However, the physical transfer function of analog devices including photonic circuits, electro-optic NLUs, modulators, and PDs cannot perfectly match the mathematical model in the digital computer, so the output of the photonic feature extractor will deviate from the supposed results. Although the transfer learning method adopted can compensate few deviations, a too-large deviation will still lead to a failure of transfer learning. The factor that majorly influences the mismatch includes weight tuning precision, frequency response of optoelectronic devices, nonideal nonlinear profile, and random noise. The way of achieving high-accuracy weight tuning is widely investigated and recent works demonstrate 9-bit precision on MRR weighting banks [S5]. Moreover, merging the measured frequency response of optoelectronic devices into the digital network model is an effective way to compensate for the deviation introduced by frequency response [S6]. Based on these pioneer investigations, we additionally evaluate the influence of the nonideal nonlinear profiles and random noise by numerical simulation.

When the nonlinear profile of the electro-optic NLU is nonideal, it introduces a fixed error to the output signal, which can be modeled as adding another deviation function on the ideal nonlinear function. In the numerical simulation, we adopt a quadratic function as the deviation function, shown as:

$$f_{\text{EO}}(x)=\text{ReLU}+f_{\text{dev}}=\max\{0,x\}+\varepsilon\cdot x^{2}$$

where *ε* measures the deviation level, i.e. “NLU error” in Figs. 4e and 4f. We replace all nonlinear activation layers in the digital network model with the nonideal nonlinear function and run inference. Results are shown in Fig. 4e, which suggests that the deviation of the nonlinear function will influence the feature maps and consequently decrease the accuracy of the classifier. Then, the classifier is retrained with the nonideal feature maps. The deviation of the nonlinear function is well compensated and the classification accuracy is retrieved.

Typically, the noise of a photonic circuit is mostly introduced by the amplified PD. To simulate the influence of random noise, we add Gaussian noise to every convolution result before the nonlinear activation. The standard deviation of the added Gaussian noise is denoted as “noise level” in Figs. 4e and 4f. The transfer learning can also compensate for low-level random noise.

**S4 Demonstration of a simplified two-layer PAFE system**

The experimental setup is depicted in Fig. S2. The two-channel input signal is generated with an AWG. The linear part of the first layer is implemented with the photonic chip. It carries out the spatiotemporal feature extraction with the kernel shape of [number of input channels × number of delay steps] = [2 × 3]. Then, a following distributed feedback (DFB) laser (Shijia, T-20-550-B-07) conducts the ReLU nonlinear activation. When the input electrical signal is larger than the threshold, the DFB laser will output optical signals, otherwise, there is no optical output. A bias voltage *V*_TH_ is applied to configure the threshold of the DFB laser. The activated feature map is sent to the second feature extraction layer which is implemented by discrete components. Its kernel shape is [1 × 3]. Attenuators (ATTs) are used for weight configuration. Also, a DFB laser follows as the ReLU activation. A low-pass filter (LPF, DC-5GHz) is adopted after the DFB laser to filter out the high-frequency relaxation spurs of the laser output. Finally, an ADC samples the extracted feature map at the speed of 1/4 of the original signals.

The input signals are two pulse trains with normalized amplitudes of [1, 2, 3, 0, 0, 0, 2, 4, 6, 0, 0, 0, 3, 6, 9] and [3, 2, 1, 0, 0, 0, 6, 4, 2, 0, 0, 0, 9, 6, 3], respectively. The time interval of the input pulses is set at 1 ns, corresponding to a speed of 1 GHz. The convolutional kernel of the first layer and the second layer is [1, 2, 3; 3, 2, 1] and [1, 2, 3], respectively. Therefore, the feature extractor will output large values when the kernel matches the input sequence. Fig. S3a and S3b show the experimental results before and after the nonlinear activation of the first layer. The reference line V_TH_ shows the bias voltage of the DFB laser. The red dots are the simulated result samples under the rule of strided convolution. Fig. S3c and S3b show the results before and after the nonlinear activation of the second layer. The experimental results are close to the simulated samples. Errors are mainly introduced from the nonideal ReLU activation, which can be compensated via the ADT-learning process. After the strided convolution, the time interval of the samples is 4 ns, corresponding to a sampling rate of 250 MSa

s^-1^, a quarter of the original signal speed.

**S5 Comparison study with different neural network configurations**

Since the behavior of neural networks is not strictly explainable, only the high classification accuracy achieved by the PAFE does not provide sufficient evidence to verify the photonic feature extractor’s effectiveness in obtaining valid spatiotemporal features. Therefore, we need a comparison study to strengthen our claim about the effectiveness of the photonic feature extractor.

Firstly, we ascertain whether the high classification accuracy is achieved only by the fully-connected classifier. A neural network with only fully connected layers is built and trained with the same dataset for pretraining the PAFE. Its number of output neurons is the same as the last two layers of experimental CNN. The training curves are shown in Fig. S4 and the classification results are shown in Fig. 4a. The accuracy of 89.8% suggests that only a fully connected classifier is not able to achieve good performance. The convolutional feature extractor is important for transforming the raw input data into classifiable features.

Secondly, the functionality of spatiotemporal feature extraction is studied. A neural network with a single input channel is built and trained. Only one channel of the raw signal is processed by the neural network so that it cannot acquire any spatial information. The structure of the network is complete with convolutional layers and fully-connected layers. The number of output channels and output neurons is the same as the one adopted in the PAFE experiment. Fig. S5 depicts the training curves and Fig. 4b shows the classification results of 91.5%. Therefore, we can conclude that the spatial-temporal-joint feature extraction is a key factor in achieving high classification accuracy.

Thirdly, we ascertain whether the analog feature extractor performs valid transformations to the raw input signal or just down-samples the input signal. A complete CNN with the same structure as adopted in the experiment is built. A dataset with a directly 4-time down-sampled raw signal is generated to train the network. Training curves are shown in Fig. S6 and classification accuracy are depicted in Fig. 4c. Results suggest that directly down-sampling will damage the information contained in the raw signal. However, with the photonic feature extractor, valid information is maintained although the sampling rate is reduced.

From the results of the above comparison study, we verify that photonic spatiotemporal feature extraction is the fundamental reason for high classification accuracy. The effectiveness of the PAFE concept is thus strengthened.

**Supplementary References**

[S1] A. Zhang, J. Li, L. Feng, K. Lv, F. Yan, Y. Yang, H. Wang, Q. Yang, L. Wang, X. Zhang, S. Ding, M. Liao, Y. Yu, and L. Li, Field trial of 24-Tb/s (60 × 400Gb/s) DWDM transmission over a 1910-km G.654.E fiber link with 6-THz-bandwidth C-band EDFAs, Optics Express **29**, 43811-43818 (2021).

[S2] A. N. Tait, Silicon photonic neural networks, Ph.D dissertation at Princeton university, 2018.

[S3] S. Xu, J. Wang, and W. Zou, Optical convolutional neural network with WDM-based optical patching and microring weighting banks, IEEE Photonics Technology Letters **33**, 89-92 (2021).

[S4] T. Ferreira de Lima, A. N. Tait, H. Saeidi, M. A. Nahmias, H. Peng, S. Abbaslou, B. J. Shastri, and P. R. Prucnal, Noise analysis of photonic modulator neurons, IEEE Journal of Selected Topics in Quantum Electronics **26**, 7600109 (2020).

[S5] W. Zhang, C. Huang, H. Peng, S. Bilodeau, A. Jha, E. Blow, T. Ferreira de Lima, B. J. Shastri, and P. Prucnal, Silicon microring synapses enable photonic deep learning beyond 9-bit precision, Optica **9**, 579-584 (2022).

[S6] G. Mourgias-Alexanderis, M. Moralis-Legios, A. Tsakyridis, N. Passlis, M. Kirtas, A. Tefas, T. Rutirawut, F. Y. Gardes, and N. Pleros, Channel response-aware photonic neural network accelerators for high-speed inference through bandwidth-limited optics, Optics Express 30, 10664-10671 (2022).

**Fig. S1 Schematic of strided convolution in the analog domain.** **a,** Strided convolution of the first layer. **b,** Strided convolution of the second layer.

**Fig. S2** Experimental setup of the small-size two-layer PAFE system. It corresponds to the yellow-box-surrounded structure of the convolutional neural netowork. Several monitor points are marked to inspect the intermediate results.

**Fig. S3** Experimental results of the two-layer PAFE system. a-c, The intermediate results of monitor point A, point B, and point C, respectively. d, The final result of monitor point D. The red dots are the simulated sample points calculated from the input pulse train. The interval of these sample points follows the rule of strided convolution, which can be found in Supplementary note S1. The error is introduced by the nonideality of the DFB laser and relaxation oscillation.

**Fig. S4** Training curves of the comparison study, in the case that the input data is Nyquist-sampled but the neural network only contains fully connected layers.

**Fig. S5** Training curves of the comparison study, in the case that the input data is the single-channel Nyquist-sampled and the neural network is complete, containing convolutional layers and fully-connected layers.

**Fig. S6** Training curves of the comparison study, in the case that the input data is down-sampled (÷4) and the neural network is complete, containing convolutional layers and fully-connected layers. Since an obvious overfitting occurs, the highest classification accuracy around 80-epoch is chosen for the comparison in Fig. 4d.

**Fig. S7** **Optical spectrum of the single-sideband carrier-suppressed modulation.** The carrier and the lower sideband are suppressed over 20 dB below the upper sideband.

**Fig. S8 Ray tracing simulation for generating the pretraining dataset.** a-d, examples of ray tracing of different targets, “I” “M” “L” and “C”, respectively. As long as the reflected rays enter the reception aperture of the receiving antennas (RAs), the rays are regarded as sub-echoes.

**Fig. S9 The dataset of pretraining.** Randomly picked 40 examples are shown. The red line shows the echo of receiving antenna 1 and the blue line shows the echo of receiving antenna 2. Corresponding labels are marked for each subfigure.
